# Physic, Law, and Drunkenness

**Published:** 1890-03-08

**Authors:** 


### The Hospital

A Weekly Journal of

### Science, Medicine, Nursing, and Philanthropy.

*For Hospitals, Asylums, Medical Practitioners, Students, Nurses, Families, Parishes, Congregations, and the Charitable Public.*

Vol. VII.—No. 180.] [March 8, 1890.

### PHYSIC, LAW, AND DRUNKENNESS.

Professor W. T. Gairdner, of Glasgow, who is also Physician in Ordinary to the Queen for Scotland, may be accepted as representing all that is wisest and best in philanthropic medicine on the question of habitual drunkenness. Dr. Gairdner is no ordinary medical practitioner. As a physician he includes in himself whatever has been proved worthiest both in the old and new schools of medical thought and practice: as a man of broad and liberal culture he has few equals; as a philanthropist of deep religious earnestness, of life-long experience, and of sober sense, he has no superior in the medical profession, nor perhaps out of it. In Scotland, where Professor Gairdner is best known, his judgments on important public questions are held to be of great value. Lawyers and judges, who do not err in excess of reverence for medical opinion, pay Dr. Gairdner's utterances the respect of earnest consideration; politicians and the general public who have ordinarily no more regard for medical oracles than lawyers and judges, exempt Dr. Gairdner from the class of practitioners whose opinions are of no particular importance to anyone. We have reasons for being at some pains to explain to English, and more particularly to Metropolitan readers the peculiar eminence to which Dr. Gairdner has attained in the minds of his fellow countrymen by a long life conspicuous for ability, fairness, and public spirit.

The opinions recently published by Professor Gairdner on the question of the legal management of habitual drunkards, and which ought rather to be called "convictions" than mere opinions, demand sober consideration at the hands of all who take interest in this important practical question. What ought we to do with the man or woman who habitually gets drunk? What ought to be done to that trade and those traders who encourage habitual drunkenness? What will the law of England permit us to do? What ought it to permit us to do? These are questions that should be capable of ready answer. For a civilised community like our own, which has a legal system at least a thousand years old, and a venerable religion much older still, ought to be able to deal in some reasonably efficient fashion with every urgent social problem that presents itself for solution.

No open-minded person, least of all anyone who has had experience of habitual drunkards, will deny that the question of their management is both important and urgent. Dr. Gairdner shows that so long ago as 1593 Sir Edward Coke, then Speaker of the House of Commons, and the greatest lawyer of his time, had come to the conclusion that the habitual drunkard is, to all intents and purposes, an insane person. In Coke's own words he is to be considered as "voluntarius dæmon," or possessed and dominated by an evil spirit, not his own, but whom he voluntarily invites to take possession of him. Bacon, who had no special desire to agree with Coke, held similar opinions. It is probable that most of the able English and Scotch lawyers who have lived since the days of Bacon and Coke, have been entirely at one with them as to the practical insanity of drunken persons. On a subject of this nature scientific medical opinion is of even more value than legal. That a person profoundly under the influence of alcoholic drink is for all practical purposes of unsound mind no competent medical man will for a moment doubt: that a man who is habitually drunk is *ipso facto* habitually insane follows as an obvious corollary. There is, indeed, no necessity to demonstrate so very self-evident a fact. The only reason for quoting distinguished scientific opinion is that private judgment may be sustained in its conclusions. In every-day life all classes of persons act upon the implicit assumption that a person in drink is a person bereft of his reason for the time.

But whilst everybody is agreed that drunkards come under the class of insane persons, and that insane persons ought to be managed in a certain particular way: and whilst the law has embodied general opinion in compulsory statutes so far as ordinary insane persons are concerned, all varieties of drunkards are exempted from the action of those statutes. It is not creditable to English Jurisprudence that it fails to provide an efficient method of dealing with habitual drunkards. If there were any doubt on the question of the practical insanity of such persons, or if that insanity were of such a kind as to be entirely harmless to others, the state of the law on the point would be of little moment. But if the question be fairly considered it will have to be admitted that no single form of insanity produces more widespread injury and misery than habitual drunkenness. The insane person of homicidal or suicidal tendency is promptly placed under restraint, to his own advantage and that of others. But the habitual drunkard may destroy both his health and his character, ruin his home, bring his family to beggary, and end by killing others or himself. Yet until he does some act which the law recognises as criminal there is nothing to prevent him from continuing his reckless and dangerous conduct for a whole life-time. As Dr. Gairdner says, "Up to the present time John Bull may be said to have set up his back, and practically to hold the field against all comers, in the name of the 'liberty of the subject.'" That, as all the initiated know too well, is what really blocks the way against further legislation. Stated in a few words, it really means this: that the habitual drunkard, in order that he may retain his own liberty, shall have full liberty to destroy not only the liberty, but also the very means of subsistence of all who are dependent upon him.

Dr. Gairdner, and of course all who agree with him, that is practically all who have studied the question, demand legislation of a compulsory character. As everybody knows, the "Habitual Drunkards Act, 1879," is purely permissive, and being permissive it is practically worthless. It is quite impossible for any ordinary logic to show why habitual drunkards, whom both lawyers, doctors, statesmen, and the general public admit to be insane, and not only insane, but dangerous, should be at full liberty to please themselves whether they will be placed under restraint or not, whilst all other persons who are insane only, without being dangerous, may be placed under restraint without any reference to their own wish in the matter. In the domain of argument the battle of compulsion has been won over and over again.

The real practical difficulty, it seems to us, is overlooked by the compulsionists. It consists in this, that Parliament has not time for the consideration of such a comparatively insignificant question as the management of habitual drunkards. The subject is not really insignificant in itself, but by comparison with the large number of legislative questions which interest hundreds, whilst the drunkard's difficulty concerns but units, it is not of overwhelming magnitude. It cannot be expected that Parliament will leave the discussion of the more urgent and interesting affairs for that of the less urgent and interesting. May we not in this dilemma take a leaf out of the book of those who carried the "Compulsory Notification of Infectious Diseases Act" safely through the deep waters? That Act, so far as Parliament is concerned, is both permissive and compulsory. The actual responsibility is, in fact, shifted from the shoulders of the Legislature to those of the Municipality or the County Council. Those bodies are undoubtedly the proper authorities for dealing with habitual drunkards. It is probable that if a Bill were introduced empowering County Councils and Municipalities to deal with such persons compulsorily on certain prescribed lines, and if the adoption of such a measure were left to the free choice of each local authority, the Bill would be passed without difficulty or delay. All legislation on "drink" questions is tending in the direction indicated, and it is difficult to see how any generally effective measures could be taken at all except by local authorities, even if the Act of 1879 were compulsory instead of merely permissive. For our part, we deeply sympathise with Professor Gairdner and those who are acting with him, but we cannot help feeling that if any progress is to be made they must consider what is immediately practicable, and shape their measures to suit all the exigencies of the Parliamentary situation.

